# Multimorbidity patterns with K-means nonhierarchical cluster analysis

**DOI:** 10.1186/s12875-018-0790-x

**Published:** 2018-07-03

**Authors:** Concepción Violán, Albert Roso-Llorach, Quintí Foguet-Boreu, Marina Guisado-Clavero, Mariona Pons-Vigués, Enriqueta Pujol-Ribera, Jose M. Valderas

**Affiliations:** 1 Institut Universitari d’Investigació en Atenció Primària Jordi Gol (IDIAP Jordi Gol), Gran Via Corts Catalanes, 587 àtic, 08007 Barcelona, Spain; 2 Universitat Autònoma de Barcelona, Bellaterra, Cerdanyola del Vallès, Spain; 3 Department of Psychiatry, Vic University Hospital, Francesc Pla el Vigatà, 1, 08500 Vic, Barcelona Spain; 4 Faculty of Nursing, University of Girona, Emili Grahit, 77, 17071 Girona, Spain; 5 Health Services & Policy Research Group, Academic Collaboration for Primary Care, University of Exeter Medical School, Exeter, EX1 2LU UK

**Keywords:** Multimorbidity, Cluster analysis, Multiple correspondence analysis, K-means clustering, Primary health care, Electronic health records, Diseases

## Abstract

**Background:**

The purpose of this study was to ascertain multimorbidity patterns using a non-hierarchical cluster analysis in adult primary patients with multimorbidity attended in primary care centers in Catalonia.

**Methods:**

Cross-sectional study using electronic health records from 523,656 patients, aged 45–64 years in 274 primary health care teams in 2010 in Catalonia, Spain. Data were provided by the Information System for the Development of Research in Primary Care (SIDIAP), a population database. Diagnoses were extracted using 241 blocks of diseases (International Classification of Diseases, version 10). Multimorbidity patterns were identified using two steps: 1) multiple correspondence analysis and 2) k-means clustering. Analysis was stratified by sex.

**Results:**

The 408,994 patients who met multimorbidity criteria were included in the analysis (mean age, 54.2 years [Standard deviation, SD: 5.8], 53.3% women). Six multimorbidity patterns were obtained for each sex; the three most prevalent included 68% of the women and 66% of the men, respectively. The top cluster included coincident diseases in both men and women: Metabolic disorders, Hypertensive diseases, Mental and behavioural disorders due to psychoactive substance use, Other dorsopathies, and Other soft tissue disorders.

**Conclusion:**

Non-hierarchical cluster analysis identified multimorbidity patterns consistent with clinical practice, identifying phenotypic subgroups of patients.

**Electronic supplementary material:**

The online version of this article (10.1186/s12875-018-0790-x) contains supplementary material, which is available to authorized users.

## Background

In the first decade of the twenty-first century, tremendous effort was concentrated on surfacing data about multimorbidity patterns in order to increase the knowledge of how the diseases were clustered [1–3]. In everyday primary care settings, multimorbidity is more the norm than an exception, with a prevalence ranging from 13 to 95% in the global population, depending on the age group included and methodology used [2]. Therefore, establishing these clustered associations could inform Clinical Practice Guidelines (CPG) and guide decision-making in the clinical practice [4].

No consensus has been established about a standard model to determine multimorbidity patterns. Differences between studies have been observed, such as the unit of analysis selected (patients versus diseases), the statistical method for grouping diseases (factor analysis vs. cluster analysis), diseases included (chronic or all), and number of diseases included in the models [1, 5].

To identify the multimorbidity patterns, methods that identify and separate certain population groups from others and study non-random associations between diseases in those sub-groups are needed [3, 6]. There are basically two statistical methods for grouping diseases: factor analysis and cluster analysis. Exploratory factor analysis is based on correlations between diagnoses to identify the patterns; it is used to test hypothesised relationships between observed measures and latent constructs and allows the inclusion of a diagnosis in multiple factors. In contrast, cluster analysis obtains the patterns of multimorbidity based on dissimilarities between diseases; clusters tend to contain diagnoses that are similar to each other (in terms of Euclidean distances) and a diagnosis cannot be included in more than one cluster. Usually, factor analysis is used to study diseases and cluster analysis to study patients [7]. A recent comparison of the two methods concluded that cluster analysis is more useful than factor analysis for in-depth study of multimorbidity patterns [8].

Among cluster analysis methods, there are two main types of techniques: hierarchical (HCA) and non-hierarchical cluster analysis (NHCA) [9]. The first, often considered when choosing a clustering technique in biomedicine, attempts to identify relatively homogeneous groups of cases based on selected characteristics, using an algorithm that either agglomerates or divides entities to form clusters. HCA is organized so that one cluster can be entirely contained within another cluster, but no other kind of overlap between clusters is allowed. However, the technique is not particularly good when it comes to robust identification of patterns in data. The main limitations are that the hierarchical clusters are susceptible to outliers in the data, the final solution depends on the chosen distance measure, and the algorithms are not efficient to analyse large data sets, as they require a large distance matrix. Nevertheless, almost all studies to date have used HCA to analyse multimorbidity patterns [2, 3].

Among the NHCA methods, K-means is the most frequently used. In contrast to HCA, this approach does not involve the construction of groups via iterative division or clustering; instead, patients are assigned to clusters once the number of clusters is specified. The results are less susceptible to outliers in the data, to the influence of choosing a distance measure, or to the inclusion of inappropriate or irrelevant variables. Algorithms that do not require a distance matrix, such as k-means, can analyse extremely large data sets [9–11].

The study of biological heterogeneity requires the identification of subgroups of populations with specific combinations of coexisting diseases. This “multimorbidity patient” approach identifies phenotypes of the subgroups, describes the patterns of diseases within each one, and facilitates the development of more targeted patient management [12].

The purpose of this study was to obtain the multimorbidity patterns in adult patients with multimorbidity attended in primary care in Catalonia (Spain), stratified by sex, using a k-means cluster analysis.

## Methods

### Design, setting and study population

A cross-sectional study was conducted in Catalonia (Spain), a Mediterranean region with 7,434,632 inhabitants, 81% of which live in urban municipalities (2010 census). The Spanish National Health Service (NHS) provides universal coverage, financed mainly by tax revenue. The Catalan Health Institute (CHI) manages primary health care teams (PHCTs) that serve 5,501,784 patients (274 PHCT), or 74% of the population; the remaining PHCTs are managed by other providers.

The CHI’s Information System for the Development of Primary Care Research (SIDIAP) contains the coded clinical information recorded in electronic health records (EHR) by its 274 PHCTs since 2006. A subset of SIDIAP records meeting the highest quality criteria for clinical data, the SIDIAP-Q, includes 1,833,125 patients attended by the 1365 general practitioners (GPs). SIDIAP Q represents 40% of the SIDIAP population whose data recording scores contain information on the majority of the population of Catalonia, and is highly representative of the whole region in terms of geography, age, sex, and diseases. This study was limited to SIDIAP-Q, as the sample was representative of the population [13].

Prevalence of individual conditions, multimorbidity, and disease patterns varies by age. To obtain a more homogenous sample of multimorbidity, we identified 408,944 patients with multimoribidity aged 45 to 64 years [14] on 31 December 2010 (Additional file 1).

### Coding and selection of diseases

Diseases are coded in SIDIAP using International Classification of Diseases version 10 (ICD-10) [15]. For this study, we selected all active diagnoses recorded in EHR as of December 31, 2010, except for R codes (symptoms, signs, and abnormal clinical and laboratory findings, not elsewhere classified) and Z codes (factors influencing health status and contact with health services). Of the 263 blocks of diagnosis in the ICD-10, excluding the R codes and Z codes yielded 241 blocks. Non-active diagnoses, based on the presence of an end date in the EHR, were excluded. These diagnoses covered a broad list of acute diseases for which the system automatically assigns an end date (e.g., 60 days after the initial diagnosis).

To facilitate information management, the diagnoses were extracted using the 263 blocks (disease categories) in the ICD-10 structure. These are homogeneous categories of very closely related specific diagnoses. For example, *Hypertensive diseases* include Essential (primary) hypertension, Hypertensive heart disease, Hypertensive renal disease, Hypertensive heart and renal disease, and Secondary hypertension. To obtain consistent and clinically interpretable patterns of association, and to avoid spurious relationships that could bias the results, we considered only diagnoses with greater than 1% prevalence in each sex. All patients with multimorbidity were included.

### Multimorbidity definition

Multimorbidity was defined by the presence of two or more ICD-10 diagnoses in the EHR from the 241 blocks selected.

### Variables

The unit of measurement was the diagnoses included in the 241 blocks (disease categories) of the ICD-10 structure (values: 1 if present, 0 if absent). Other variables recorded were number of diseases, age (in years), and sex (women, men).

No missing values were handled, as sex and age were recorded for all patients. Wrong sex-specific diagnosis codes and diagnoses with inconsistent dates were excluded during data cleaning. Any record with no disease diagnoses was considered as a disease-free individual.

### Statistical analysis

Analyses were stratified by sex. Descriptive statistics were used to summarize overall information. Categorical variables were expressed as frequencies (percentage) and continuous variables as mean (Standard deviation, SD) or median (interquartile range, IQR). Two sample tests of proportions were used to assess sex-based differences between groups Mann Whitney was used to test the non-normally distributed variable of number of blocks of diagnoses by sex.

We identified disease patterns using two steps:

1. Multiple Correspondence Analysis (MCA): A data analysis technique for nominal categorical data, was used to detect and represent underlying structures in the data set. The method allows representation in a multidimensional space of relationships between a set of dichotomous or categorical variables (in our case, diagnoses) that would otherwise be difficult to observe in contingency tables and show groups of patients with the same characteristics [16]. MCA also allows direct representation of patients as points (coordinates) in geometric space, transforming the original binary data to continuous data (Additional file 2). The MCA analysis was based on the indicator matrix. Optimal number of dimensions extracted and percentages of inertia were determined by the means of scree plot.
2. K-means clustering: From the geometric space created in MCA, patients were classified into clusters according to proximity criteria by means of the k-means algorithm. The algorithm is composed of the following steps: 1) Place K points into the space represented by the patients that are being clustered. These points represent initial group centroids. 2) Assign each patient to the group that has the closest centroid. 3) When all patients have been assigned, recalculate the positions of the K centroids. Repeat Steps 2 and 3 until the centroids no longer move. This produces a separation of the patients into homogenous groups while maximizing heterogeneity across groups [9]. The optimal number of clusters is the solution with the highest Calinski-Harabasz index value. To assess internal cluster quality, cluster stability of the optimal solution was computed using Jaccard bootstrap values with 100 runs [17]. Highly stable clusters should yield average Jaccard similarities of 0.85 and above [9].

#### Statistics of multimorbidity patterns

To describe the multimorbidity patterns in patients, frequencies and percentages of diseases in each cluster were calculated. Observed/expected ratios (“O/E-ratios”) were calculated by dividing disease prevalence in the cluster by disease prevalence in the sex group. A disease was considered to be associated with the multimorbidity pattern when O/E-ratio was ≥2 [18]. Exclusivity, defined as the fraction of patients with the disease included in the cluster over the total strata patients with the disease, was also calculated. To describe the relative position of the clusters, centrality defined as the distance of the cluster centroid to the origin was calculated. Descriptive statistics of age and the median number of diagnoses for each cluster were also obtained. Clinical criteria were used to evaluate the consistency and utility of the final cluster solution. To reduce the size of the tables, only groups of diseases with a prevalence higher than 10% in the cluster were shown.

The analyses were carried out using SPSS for Windows, version 18 (SPSS Inc., Chicago, IL, USA) and R version 3.3.1 (R Foundation for Statistical Computing, Vienna, Austria).

## Results

Out of 523,656 patients aged 45 to 64 years, 408,994 (78.1%) met the multimorbidity criteria. Women had a higher multimorbidity prevalence than men (82.2% vs. 73.9%, *p* < 0.001). The mean age was 54.2 years (Standard deviation [SD]: 5.8), 53.3% were women, and the mean number of diagnoses per patient was 5.7 (SD: 3.3). The analysis included 217,823 women and 191,171 men with 79 and 73 different diagnoses, respectively (Table 1 and Additional file 3).

**Table 1:** Number of diseases for patients 45–64 years old, stratified by sex, Catalonia, 2010*

	Women n (%) 217,823 (82.2)	Men n (%) 191,171 (73.9)
Number of diagnoses^†^		
2	26,106 (12.0)	33,850 (17.7)
3	28,243 (13.0)	33,515 (17.5)
4	28,274 (13.0)	30,356 (15.9)
≥ 5	135,200 (62.1)	93,450 (48.9)
Median number of diagnoses (IQR)^‡^	5 (4–8)	4 (3–7)
Number of diagnoses included	79	73

Abbreviations: *IQR* inter-quartile range

*Included in the analysis *N* = 523,656, people with ≥2 diagnoses; 408,994 (78.1%)

^†^Two sample test of proportions; all *p*-values< 0.001

^‡^Mann-Whitney test; *P* < 0.001

**Fig. 1:**
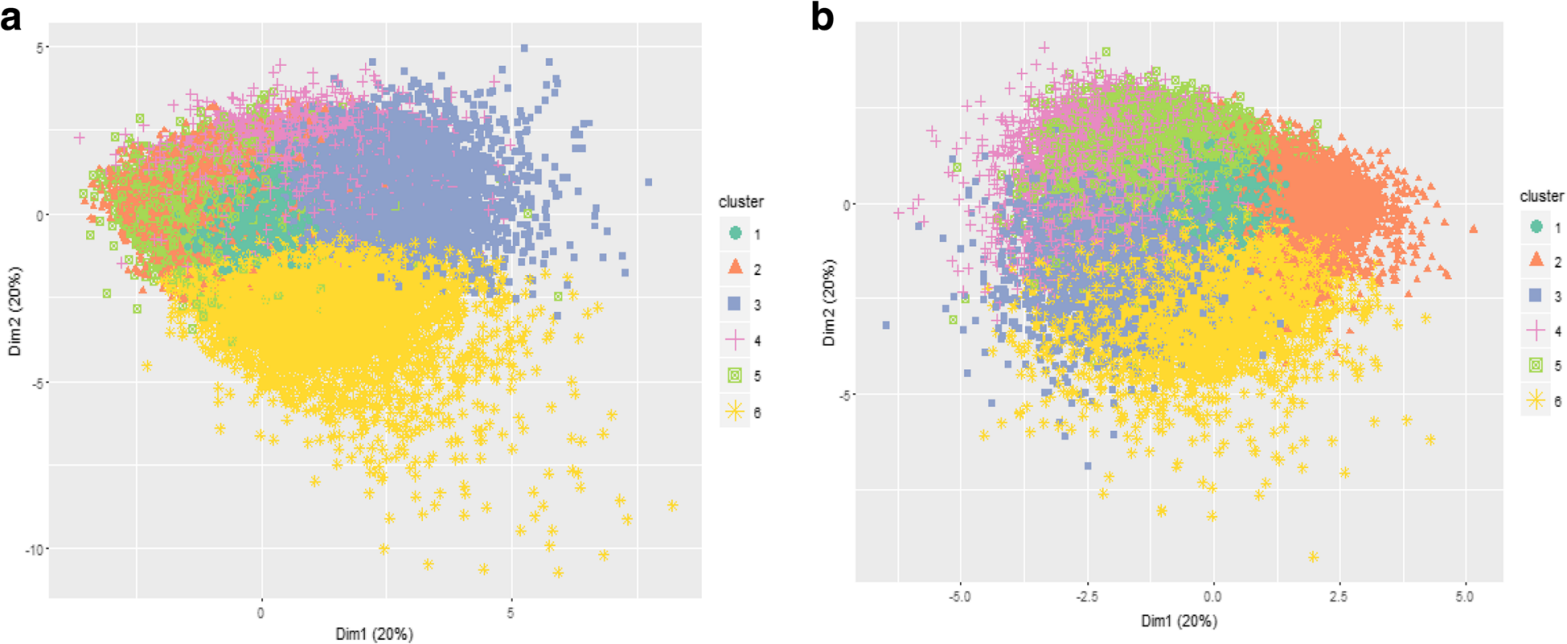
**a** and **b**. Patients cluster plot for women (*n* = 217,823) and men (*n* = 191,171) aged 45–64 years, analysed with k-means clustering

Data were transformed using MCA (Additional file 2). K-means clustering using Calinski criterion to obtain six clusters was considered the optimal solution for both women and men. Average Jaccard bootstrap values for women and men were 0.98 and 0.90, respectively, showing highly stable solutions. A spatial representation of clusters is shown with a cluster plot for women (Fig. 1a) and men (Fig. 1b).

Six multimorbidity patterns were obtained for each sex. The three most prevalent multimorbidity patterns included 68.4% of women patients (Table 2) and 65.6% of men patients (Table 3). The number of diseases included in each pattern varied by sex; women had a higher number of diseases than men, although there was a high coincidence (matching) between them in the type of diseases grouped.

**Table 2:** Three most prevalent multimorbidity patterns in women (*n* = 217,823) aged 45–65 years, Catalonia, 2010

Cluster n (%)^a^	Blocks of diagnoses	Prevalence in cluster (%)^b^	Prevalence in women (%)^c^	O/E ratio^d^	Exclusivity (%)	Centrality	Mean Age	Median number of diagnoses
**1**	E70-E90:Metabolic disorders	25.9	35.4	0.73	29.8	0.8	53.0	3
88,657 (40.7)	M50-M54:Other dorsopathies	23.6	35.8	0.66	26.9			
	F10-F19:Mental and behavioural disorders due to psychoactive substance use	21.1	18.6	1.13	46.1			
	F40-F48:Neurotic. stress-related and somatoform disorders	20.0	27.3	0.73	29.9			
	N80-N98:Noninflammatory disorders of female genital tract	17.6	24.2	0.73	29.6			
	I10-I15:Hypertensive diseases	15.6	25.6	0.61	24.9			
	M70-M79:Other soft tissue disorders	13.9	27.0	0.52	21.0			
	E00-E07:Disorders of thyroid gland	11.8	14.9	0.79	32.3			
	D10-D36:Benign neoplasms	10.4	16.2	0.65	26.3			
**2**	M50-M54:Other dorsopathies	55.4	35.8	1.55	23.0	1.6	57.4	7
32,249 (14.8)	E70-E90:Metabolic disorders	53.6	35.4	1.52	22.4			
	**M15-M19:Arthrosis**	**48.2**	**15.7**	**3.08**	**45.6**			
	M70-M79:Other soft tissue disorders	47.5	27.0	1.76	26.1			
	**M80-M85:Disorders of bone density and structure**	**38.7**	**11.3**	**3.41**	**50.5**			
	M20-M25:Other joint disorders	33.0	18.6	1.78	26.3			
	F40-F48:Neurotic. stress-related and somatoform disorders	30.1	27.3	1.10	16.3			
	I10-I15:Hypertensive diseases	29.3	25.6	1.14	16.9			
	I80-I89:Diseases of veins. Lymphatic vessels and lymph nodes. Not elsewhere classified	29.2	18.3	1.60	23.7			
	F30-F39:Mood [affective] disorders	20.8	14.6	1.43	21.1			
	N80-N98:Noninflammatory disorders of female genital tract	20.5	24.2	0.85	12.5			
	E65-E68:Obesity and other hyperalimentation	20.5	19.0	1.08	16.0			
	**G50-G59:Nerve. nerve root and plexus disorders**	**20.0**	**8.5**	**2.34**	**34.7**			
	**M45-M49:Spondylopathies**	**19.7**	**4.3**	**4.56**	**67.4**			
	E00-E07:Disorders of thyroid gland	17.8	14.9	1.20	17.7			
	**M40-M43:Deforming dorsopathies**	**15.1**	**3.8**	**3.96**	**58.6**			
	D10-D36:Benign neoplasms	12.4	16.2	0.77	11.4			
	K20-K31:Diseases of oesophagus. Stomach and duodenum	12.0	11.4	1.05	15.6			
	J30-J39:Other diseases of upper respiratory tract	11.2	9.4	1.19	17.6			
	G40-G47:Episodic and paroxysmal disorders	11.2	10.5	1.06	15.7			
	J00-J06:Acute upper respiratory infections	11.1	12.6	0.88	13.0			
	H90-H95:Other disorders of ear	10.2	6.3	1.60	23.7			
**3**	N80-N98:Noninflammatory disorders of female genital tract	48.1	24.2	1.99	25.6	1.7	53.0	8
28,024 (12.9)	M50-M54:Other dorsopathies	46.9	35.8	1.31	16.9			
	M70-M79:Other soft tissue disorders	38.8	27.0	1.44	18.5			
	M20-M25:Other joint disorders	33.6	18.6	1.81	23.3			
	**D10-D36:Benign neoplasms**	**32.8**	**16.2**	**2.03**	**26.1**			
	I80-I89:Diseases of veins. Lymphatic vessels and lymph nodes. Not elsewhere classified	29.3	18.3	1.60	20.6			
	**L20-L30:Dermatitis and eczema**	**28.4**	**9.3**	**3.05**	**39.2**			
	E70-E90:Metabolic disorders	27.7	35.4	0.78	10.1			
	F40-F48:Neurotic. stress-related and somatoform disorders	26.3	27.3	0.96	12.4			
	K00-K14:Diseases of oral cavity. Salivary glands and jaws	23.3	12.1	1.93	24.9			
	**B35-B49:Mycoses**	**19.8**	**5.7**	**3.46**	**44.5**			
	**D50-D53:Nutritional anaemias**	**19.7**	**8.3**	**2.38**	**30.6**			
	**N60-N64:Disorders of breast**	**19.2**	**7.5**	**2.56**	**32.9**			
	J00-J06:Acute upper respiratory infections	16.9	12.6	1.34	17.2			
	**H53-H54:Visual disturbances and blindness**	**16.8**	**4.4**	**3.84**	**49.4**			
	E00-E07:Disorders of thyroid gland	16.7	14.9	1.13	14.5			
	**L60-L75:Disorders of skin appendages**	**16.7**	**4.8**	**3.51**	**45.2**			
	I10-I15:Hypertensive diseases	15.9	25.6	0.62	8.0			
	E65-E68:Obesity and other hyperalimentation	15.4	19.0	0.81	10.4			
	J30-J39:Other diseases of upper respiratory tract	15.2	9.4	1.61	20.8			
	G40-G47:Episodic and paroxysmal disorders	14.0	10.5	1.33	17.1			
	**B00-B09:Viral infections characterized by skin and mucous membrane lesions**	**13.9**	**4.3**	**3.21**	**41.2**			
	**H90-H95:Other disorders of ear**	**12.9**	**6.3**	**2.03**	**26.2**			
	**H49-H52:Disorders of ocular muscles. Binocular movement. Accommodation and refraction**	**12.9**	**3.5**	**3.64**	**46.8**			
	**L80-L99:Other disorders of the skin and subcutaneous tissue**	**12.8**	**3.3**	**3.83**	**49.3**			
	**H10-H13:Disorders of conjunctiva**	**12.2**	**3.8**	**3.21**	**41.3**			
	F30-F39:Mood [affective] disorders	11.7	14.6	0.80	10.3			
	K55-K63:Other diseases of intestines	11.3	8.6	1.32	17.0			
	M15-M19:Arthrosis	11.1	15.7	0.71	9.2			
	K20-K31:Diseases of oesophagus. Stomach and duodenum	10.3	11.4	0.91	11.7			

^a^Individuals (% of total women) / ^b^Individuals as % of cluster / ^c^Individuals as % of total women)

^d^Observed / Expected Ratio. Values ≥2 in bold

**Table 3:** Three most prevalent multimorbidity patterns in men (*n* = 191,171) aged 45–65 years, Catalonia, 2010

Cluster n (%)^a^	Blocks of diagnoses	Prevalence in cluster (%)^b^	Prevalence in men (%)^c^	O/E ratio^d^	Exclusivity (%)	Centrality	Mean Age	Median number of diagnoses
**1**	E70-E90:Metabolic disorders	38.4	42.2	0.91	35.3	0.8	53.3	3
73,979 (38.7)	I10-I15:Hypertensive diseases	28.1	32.5	0.86	33.4			
	F10-F19:Mental and behavioural disorders due to psychoactive substance use	25.4	33.6	0.76	29.2			
	M50-M54:Other dorsopathies	20.8	27.8	0.75	28.9			
	M70-M79:Other soft tissue disorders	10.7	16.9	0.63	24.6			
	E65-E68:Obesity and other hyperalimentation	10.6	14.6	0.73	28.2			
**2**	**F10-F19:Mental and behavioural disorders due to psychoactive substance use**	**77.3**	**33.6**	**2.30**	**34.9**	1.5	52.6	4
28,951 (15.1)	E70-E90:Metabolic disorders	26.4	42.2	0.63	9.5			
	F40-F48:Neurotic. stress-related and somatoform disorders	25.1	13.5	1.86	28.1			
	M50-M54:Other dorsopathies	23.7	27.8	0.85	12.9			
	K00-K14:Diseases of oral cavity. Salivary glands and jaws	23.2	12.0	1.93	29.2			
	**J40-J47:Chronic lower respiratory diseases**	**19.4**	**9.3**	**2.09**	**31.6**			
	**F30-F39:Mood [affective] disorders**	**17.0**	**6.3**	**2.72**	**41.2**			
	**B15-B19:Viral hepatitis**	**16.6**	**3.2**	**5.13**	**77.6**			
	I10-I15:Hypertensive diseases	14.2	32.5	0.44	6.6			
	**K70-K77:Diseases of liver**	**12.5**	**5.2**	**2.38**	**36.1**			
	K20-K31:Diseases of oesophagus. Stomach and duodenum	12.3	11.5	1.06	16.1			
	M70-M79:Other soft tissue disorders	10.4	16.9	0.62	9.4			
**3**	E70-E90:Metabolic disorders	43.4	42.2	1.03	12.1	1.9	55.2	6
22,458 (11.8)	**K20-K31:Diseases of oesophagus. Stomach and duodenum**	**40.0**	**11.5**	**3.47**	**40.7**			
	**K40-K46:Hernia**	**31.3**	**8.8**	**3.57**	**41.9**			
	**N40-N51:Diseases of male genital organs**	**30.9**	**12.1**	**2.54**	**29.9**			
	I10-I15:Hypertensive diseases	30.3	32.5	0.93	10.9			
	M50-M54:Other dorsopathies	29.6	27.8	1.06	12.5			
	**I80-I89:Diseases of veins. Lymphatic vessels and lymph nodes. Not elsewhere classified**	**29.6**	**10.0**	**2.95**	**34.7**			
	**K55-K63:Other diseases of intestines**	**28.2**	**6.4**	**4.39**	**51.6**			
	**D10-D36:Benign neoplasms**	**21.1**	**8.6**	**2.46**	**28.9**			
	F10-F19:Mental and behavioural disorders due to psychoactive substance use	20.8	33.6	0.62	7.3			
	F40-F48:Neurotic. stress-related and somatoform disorders	19.7	13.5	1.46	17.2			
	**J30-J39:Other diseases of upper respiratory tract**	**16.1**	**8.0**	**2.01**	**23.6**			
	M70-M79:Other soft tissue disorders	15.6	16.9	0.92	10.9			
	G40-G47:Episodic and paroxysmal disorders	13.1	7.4	1.77	20.8			
	**N20-N23:Urolithiasis**	**13.0**	**4.3**	**3.00**	**35.3**			
	J40-J47:Chronic lower respiratory diseases	12.0	9.3	1.29	15.1			
	H90-H95:Other disorders of ear	10.8	7.7	1.40	16.5			

^a^Individuals (% of total men) / ^b^Individuals as % of the cluster /^c^Individuals as % of total men

^d^Observed / Expected Ratio. Values ≥2 in bold

The clusters were sorted in descending order by number of individuals included. The first cluster included about 40% of the population (40.7% of women and 38.7% of men) and no O/E ratio higher than 2 was observed in these first clusters. In these first clusters, the highest exclusivity value was 46.1% for *Mental and behavioural disorders due to psychoactive substance use* (tobacco) in women and 35.3% for *Metabolic disorders* in men.

The most prevalent cluster included coincident diseases in both men and women: *Metabolic disorders*, *Hypertensive diseases*, *Mental and behavioural disorders due to psychoactive substance use*, *Other dorsopathies* and *Other soft tissue disorders* (Tables 2 and 3).

Four other patterns were almost coincident between the sexes: 1) Cluster 4 (women) and cluster 3 (men), composed mostly of diseases of the digestive and musculoskeletal system; 2) Cluster 2 (women) and Cluster 4 (men), connective tissue diseases; 3) Cluster 5 was composed of a cardiometabolic pattern (obesity, hypertension and diabetes) in both groups; and 4) Cluster 6, infectious and injurious diseases (see Tables 2 and 3). O/E ratios varied for each cluster, peaking at 8.99 for *Other viral diseases* and 8.24 for *Other acute lower respiratory infections* in cluster 6 (women) (Tables 2 and 3).

In both sexes, the most prevalent multimorbidity pattern in the oldest patients (Tables 2 and 3) were musculoskeletal system and connective tissue diseases in women (mean age: 57.4) and cardiometabolic pattern (obesity, hypertension, and diabetes) in men (mean age: 57.1).

Multimorbidity patterns considering only blocks of diagnoses with O/E ratio ≥ 2, ordered by exclusivity in women and men, showed that the highest exclusivity in women was observed in Cluster 6: 83.9% of the people who had a diagnosis of *Other viral diseases* are included in this cluster. They were followed by Cluster 5, which 77.0% of people with *Diabetes mellitus* belonged to. In men, 83.7% of people with *Disorders of choroid and retina* belongs to Cluster 5, and 77.6%, which includes *Viral hepatitis*, in Cluster 2 (Additional file 4). 

## Discussion

Non-hierarchical cluster analysis yielded an informative categorization of patients, generating reasonable multimorbity patterns from a clinical, practical perspective, and identified phenotypes for sub-groups of patients. Metabolic-circulatory-tobacco use-musculoskeletal pattern is the most common multimorbidity pattern identified by NHCA in both sexes. This pattern would be classified as nonspecific because it had the lowest centrality value (0.8 for both sexes). It is the most common in the population with multimorbidity aged 45–65 years. This pattern seems to be consistent with other studies which obtained similar associations of diseases with other methods of analysis [2, 3].

Other data of interest are the higher exclusivity values obtained in some clusters. For example, 77% of women who suffered diabetes mellitus have other associated diseases, such as forms of heart disease, obesity, and hypertension. These results are similar to the report from Hughes et al. that 71% of people with diabetes had multimorbidity [19]. Other coexisting diseases in the 84% of men who had disorders of choroid and retina (ischemic heart diseases, diseases of arteries, arterioles and capillaries, diabetes, other forms of heart disease, obesity, and hypertension) reflect a broad affectation of the vascular tree. Another remarkable observation in some patterns was the clustering of diseases of the same system or the presence of diseases, reflecting a complication. For example, one multimorbidity pattern consisted of seven diseases, of which five were diseases of the musculoskeletal system and connective tissue (Cluster 2, women). Another well-known example is the complications of diabetes mellitus such as disorders of choroid and retina (diabetic retinopathy) and renal failure (Cluster 5, men).

These results can be translated into clinical practice. When a disease is first diagnosed, we can suspect other associated diseases. Clinical practice guidelines could orient their recommendations toward these sub-groups (for example: arthritis, anxiety and depression). On the other hand, some results could be difficult to interpret in the context of current knowledge. Some patterns obtained included many diseases with no apparent connection between them.

In general, it is difficult to compare our results with the findings of other studies because of variations in methods, data sources and structures, populations, and diseases studied. However, there are some similarities between the current study and others. The first pattern is similar to the cardio-metabolic pattern reported by Prados et al. in adults aged 45 to 64 years (hypertension, diabetes, obesity, and lipid metabolism disorders) with an exploratory factor analysis [6]. In participants older than 50 years, another study found a cardiorespiratory factor (angina, asthma, and chronic lung disease) quite similar to our Cluster 5 in men and a mental-arthritis factor (arthritis, anxiety and depression) similar to our Cluster 2 in women [20].

The major strength of this study is the large, high-quality population database of primary care records that have been shown to be representative of a much larger population [13]. The analysis was stratified by sex and a patient-level perspective was used with NHCA. Admittedly, this analysis of almost all potential diagnoses may have added a complexity that will hinder interpretation of findings and comparison with other studies. Another major strength of this study was the operational definition of multimorbidity as the co-occurrence of multiple chronic or acute diseases [21] which allows the inclusion of the full range of diseases observed in any one patient. This is especially relevant because the boundaries between chronic and acute disease are not always clear [22, 23]. The strengths of using K-means cluster analysis is that the results are less susceptible to outliers in the data, the influence of chosen distance measure, or the inclusion of inappropriate or irrelevant variables [10]. The method can also analyse extremely large data sets as in our study, as no distance matrix is required. Some disadvantages of the method are that different solutions for each set of seed points can occur and there is no guarantee of optimal clustering [12]. To minimize this shortcoming, we tested the internal validity of our solution using bootstrap methods, and the results were highly stable (Jaccard> 0.85) [17]. In addition, the method is not efficient when a large number of potential cluster solutions are to be considered [10]; to address this limitation, we computed the optimal number using analytical indexes like Calinski Harabasz [24].

A number of limitations need to be taken into account as well. The use of MCA can produce low percentages of variation on principal axes and make it difficult to choose the number of dimensions to retain. We assumed a 5-dimension solution using the elbow rule in the scree plot to achieve the most accurate solution possible without including too many dimensions in the analysis [16]. In some clusters, an accumulative diagnosis belonging to the same chapter could be coded in multiple ways; however, use of the structure of ICD10 3-character codes that group diseases as the unit of analysis, rather than the more specific individual diagnosis, makes this improbable.

Few studies have focused on the MM patterns in patients rather than on diseases [25–27]. This methodology produced results that can be transferred to clinical practice, as they suggested that diseases are not equally associated with all phenotypes and there may be a genetic basis for patterns of multimorbidity.

Multimorbidity can present a problem for health services delivery, affecting patients, health professionals, and managers who are attempting to improve service delivery [28]. Our study offers a new methodological approach to understanding the relationships between specific diseases in individual patients, which is an essential step in improving the care of patients and health systems in organizations. Analysing patient profiles permitted the identification of subgroups of patients with different associated diseases.

This study illustrates the need to pay careful attention to the methods used to support policies and decision-making. The study results have implications for three fundamental areas of action: a) the need to change the orientation of clinical guidelines that focus on a single disease; b) the need to change health policy that is based on a disease rather than on the whole person; and c) the need to change current incentive policies that focus the health professional’s attention on a disease rather than on multimorbidity, which includes not only diseases but also drug interactions, polypharmacy and the process of patient-health professional interactions.

Future studies on the current topic are therefore recommended, with a special focus on three major issues. First, the genetic typing of these multimorbidity patterns will identify genetic confluence in these patterns. Second, the delimitation of environment factors (alimentation, physical exercise, toxicity, etc.) associated with these patterns. Third, longitudinal studies should be done to establish the order of disease onset. Finally, the influence of polypharmacy, or the use of multiple drugs, could decrease treatment efficacy and cause unexpected adverse events or even the development of other diseases [29, 30].

These findings suggest that multimorbidity patterns obtained using non-hierarchical cluster analysis identified clusters more consistent with clinical practice, identifying phenotypes of certain sub-groups of patients.

## Conclusion

Non-hierarchical cluster analysis identified multimorbidity patterns consistent with clinical practice, identifying phenotypic subgroups of patients.

## Additional files

Additional file 1: Study Flow Chart, Catalonia, 2010. (DOCX 35 kb)

Additional file 2: Patients factor map for women (right, *n* = 217,823) and men (left, *n* = 191,171) aged 45–64 years. (DOCX 1468 kb)

Additional file 3: Diagnosis blocks (ICD 10) included in the Multimorbidity patterns in women and men aged 45–65 years, Catalonia, 2010. (DOCX 26 kb)

Additional file 4: Multimorbidity patterns considering only blocks of diagnoses with Observed/Expected ratio ≥ 2, ordered by exclusivity in women and men aged 45–65 years, Catalonia, 2010. (DOCX 25 kb)

## Abbreviations

CHI: Catalan Health Institute
CPG: Clinical Practice Guidelines
EHR: Electronic Health Records
HCA: Hierarchical Clustering Analysis
ICD-10: International Classification of Diseases version 10
IQR: Interquartile Range
MCA: Multiple Correspondence Analysis
NHCA: Non-hierarchical cluster analysis
NHS: National Health Service
O/E-ratios: Observed/expected ratios
PHCTs: Primary Health Care Teams
SD: Standard Deviation
SIDIAP: Information System for the Development of Research in Primary Care

## References

[1] Marengoni A, Angleman S, Melis R, Mangialasche F, Karp A, Garmen A (2011). Aging with multimorbidity: a systematic review of the literature. Ageing Res Rev.

[2] Violan C, Foguet-Boreu Q, Flores-Mateo G, Salisbury C, Blom J, Freitag M (2014). Prevalence, determinants and patterns of multimorbidity in primary care: a systematic review of observational studies. PLoS One.

[3] Prados-Torres A, Calderon-Larranaga A, Hancco-Saavedra J, Poblador-Plou B, van den Akker M (2014). Multimorbidity patterns: a systematic review. J Clin Epidemiol.

[4] Weiss CO, Varadhan R, Puhan MA, Vickers A, Bandeen-Roche K, Boyd CM (2014). Multimorbidity and evidence generation. J Gen Intern Med.

[5] Holzer BM, Siebenhuener K, Bopp M, Minder CE (2017). Evidence-based design recommendations for prevalence studies on multimorbidity: improving comparability of estimates. Popul Health Metr.

[6] Prados-Torres A, Poblador-Plou B, Calderón-Larrañaga A, Gimeno-Feliu LA, González-Rubio F, Poncel-Falcó A (2012). Multimorbidity patterns in primary care: interactions among chronic diseases using factor analysis. PLoS One.

[7] Haregu T, Oldenburg B, Setswe G, Elliott J (2012). Perspectives, constructs and methods in the measurement of multimorbidity and comorbidity: a critical review. Internet J Epidemiol.

[8] Roso-Llorach A, Violán C, Foguet-Boreu Q, Rodriguez-Blanco T, Pons-Vigués M, Pujol-Ribera E (2018). Comparative analysis of methods for identifying multimorbidity patterns: a study of “real-world” data. BMJ Open.

[9] Everitt BS, Landau S, Leese M, Stahl D (2011). Cluster analysis.

[10] Liao M, Li Y, Kianifard F, Obi E, Arcona S (2016). Cluster analysis and its application to healthcare claims data: a study of end-stage renal disease patients who initiated hemodialysis. BMC Nephrol.

[11] Ilmarinen P, Tuomisto LE, Niemelä O, Tommola M, Haanpää J, Kankaanranta H (2017). Cluster Analysis on Longitudinal Data of Patients With Adult-Onset Asthma. J Allergy Clin Immunol Pract.

[12] Fabbri E, Zoli M, Gonzalez-Freire M, Salive ME, Studenski SA, Ferrucci L (2015). Aging and multimorbidity: new tasks, priorities, and Frontiers for integrated Gerontological and clinical research. J Am Med Dir Assoc.

[13] García-Gil MM, Hermosilla E, Prieto-Alhambra D, Fina F, Rosell M, Ramos R (2011). Construction and validation of a scoring system for the selection of high-quality data in a Spanish population primary care database (SIDIAP). Inform Prim Care.

[14] Violán C, Foguet-Boreu Q, Roso-Llorach A, Rodriguez-Blanco T, Pons-Vigués M, Pujol-Ribera E (2014). Burden of multimorbidity, socioeconomic status and use of health services across stages of life in urban areas: a cross-sectional study. BMC Public Health.

[15] World Health Organization: ICD-10 International Statistical Classification of Diseases and Related Health Problems 10th Revision Version for 2010. http://apps.who.int/classifications/apps/icd/icd10online/ . Accessed 20 Feb 2016.

[16] Sourial N, Wolfson C, Zhu B, Quail J, Fletcher J, Karunananthan S (2010). Correspondence analysis is a useful tool to uncover the relationships among categorical variables. J Clin Epidemiol.

[17] Hennig C (2007). Cluster-wise assessment of cluster stability. Computational Statistics & Data Analysis.

[18] Schäfer I, Kaduszkiewicz H, Wagner HO, Schön G, Scherer M, van den Bussche H (2014). Reducing complexity: a visualisation of multimorbidity by combining disease clusters and triads. BMC Public Health.

[19] Hughes LD, McMurdo ME, Guthrie B (2013). Guidelines for people not for diseases: the challenges of applying UK clinical guidelines to people with multimorbidity. Age Ageing.

[20] Garin N, Olaya B, Perales J, Moneta MV, Miret M, Ayuso-Mateos JL (2014). Multimorbidity patterns in a national representative sample of the Spanish adult population. PLoS One.

[21] Van den Akker M, Buntinx F, Knottnerus JA (1996). Comorbidity or multi- morbidity: what’s in a name? A review of literature. Eur J Gen Pract.

[22] O'Halloran J, Miller GC, Britt H (2004). Defining chronic conditions for primary care with ICPC-2. Fam Pract.

[23] Soler JK, Okkes I, Oskam S, Van Boven K, Zivotic P, Jevtic M (2012). Revisiting the concept of 'chronic disease' from the perspective of the episode of care model. Does the ratio of incidence to prevalence rate help us to define a problem as chronic?. Inform Prim Care.

[24] Calinski RB, Harabasz JA (1974). Dendrite method for cluster analysis. Comm Stat.

[25] Guisado-Clavero M, Roso-Llorach A, López-Jimenez T, Pons-Vigués M, Foguet-Boreu Q, Muñoz MA, Violán C (2018). Multimorbidity patterns in the elderly: a prospective cohort study with cluster analysis. BMC Geriatr.

[26] Newcomer SR, Steiner JF, Bayliss EA (2011). Identifying subgroups of complex patients with cluster analysis. Am J Manag Care.

[27] Goldstein G, Luther JF, Jacoby AM, Haas GL, Gordon AJ (2008). A taxonomy of medical comorbidity for veterans who are homeless. J Health Care Poor Underserved.

[28] McPhail SM (2016). Multimorbidity in chronic disease: impact on health care resources and costs. Risk Manag Healthc Policy.

[29] Marengoni A, Onder G (2015). Guidelines, polypharmacy, and drug-drug interactions in patients with multimorbidity. Br Med J.

[30] Maher RL, Hanlon J, Hajjar ER (2014). Clinical consequences of polypharmacy in elderly. Expert Opin Drug Saf.

