# Supplementary material for: Multimorbidity patterns with K-means nonhierarchical cluster analysis
Source: BMC Fam Pract. 2018 Jul 3;19:108. doi: 10.1186/s12875-018-0790-x (PMC6031109; doi:10.1186/s12875-018-0790-x)
Supplement: Supplementary file 1 — Study Flow Chart, Catalonia, 2010. (DOCX 35 kb) [file 12875_2018_790_MOESM1_ESM.docx]

**Additional file 1. Study Flow Chart, Catalonia, 2010**

Abbreviations: SIDIAP-Q, Information System for the Development of Research in Primary Care-Quality
