# Supplementary material for: Multimorbidity patterns with K-means nonhierarchical cluster analysis
Source: BMC Fam Pract. 2018 Jul 3;19:108. doi: 10.1186/s12875-018-0790-x (PMC6031109; doi:10.1186/s12875-018-0790-x)
Supplement: Supplementary file 2 — Patients factor map for women (right, n = 217,823) and men (left, n = 191,171) aged 45–64 years. (DOCX 1468 kb) [file 12875_2018_790_MOESM2_ESM.docx]

**Additional file 2. Patients factor map for women (right, n= 217,823) and men (left, n= 191,171) aged 45-64 years.**

| **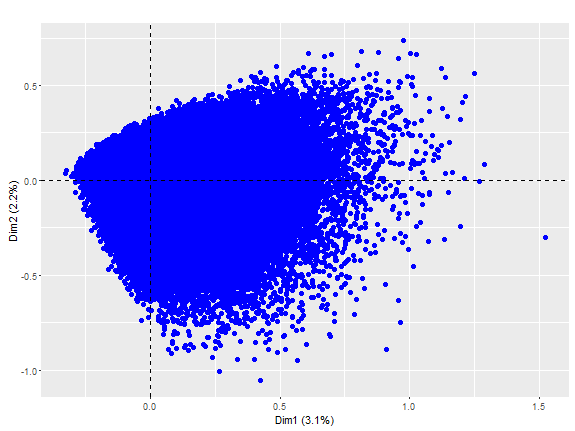** | **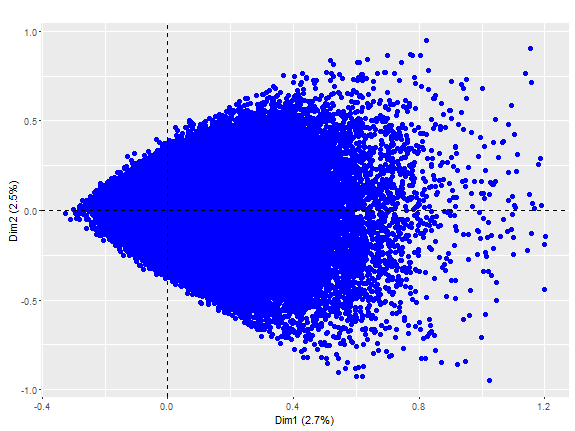** |
| --- | --- |

Notes: Analysed with multiple correspondence analysis.

Dimension (Dim) 1 and Dim 2 are the two first dimensions in the Multiple Correspondence Analysis.

We selected five as the number of components retained in MCA in each sex, accounting for a total percentage of inertia of 10.2% (women) and 10.4% (men).
