# Supplementary material for: Multimorbidity patterns with K-means nonhierarchical cluster analysis
Source: BMC Fam Pract. 2018 Jul 3;19:108. doi: 10.1186/s12875-018-0790-x (PMC6031109; doi:10.1186/s12875-018-0790-x)
Supplement: Supplementary file 3 — Diagnosis blocks (ICD 10) included in the Multimorbidity patterns in women and men aged 45–65 years, Catalonia, 2010. (DOCX 26 kb) [file 12875_2018_790_MOESM3_ESM.docx]

**Additional file 3. Blocks of diagnoses (ICD 10) included in the Multimorbidity patterns in women and men aged 45-65 years, Catalonia, 2010**

Note: all diagnoses are in the International Classification of Diseases version 10 (ICD-10) and listed in alphabetical order.
