# Supplementary material for: Multimorbidity patterns with K-means nonhierarchical cluster analysis
Source: BMC Fam Pract. 2018 Jul 3;19:108. doi: 10.1186/s12875-018-0790-x (PMC6031109; doi:10.1186/s12875-018-0790-x)
Supplement: Supplementary file 4 — Multimorbidity patterns considering only blocks of diagnoses with Observed/Expected ratio ≥ 2, ordered by exclusivity in women and men aged 45–65 years, Catalonia, 2010. (DOCX 25 kb) [file 12875_2018_790_MOESM4_ESM.docx]

**Additional file 4. Multimorbidity patterns considering only blocks of diagnoses with Observed/Expected ratio ≥ 2, ordered by exclusivity in women and men aged 45-65 years, Catalonia, 2010**

| **Women** | | | | | **Men** | | |
| --- | --- | --- | --- | --- | --- | --- | --- |
| **Cluster number** | **Blocks of diagnoses** | **Exclusivity (%)** | **Centrality** | | **Blocks of diagnoses** | **Exclusivity (%)** | **Centrality** |
|  |  |  |  | |  |  |  |
| **2** | M45-M49:Spondylopathies | 67.4 | 1.6 | | B15-B19:Viral hepatitis | 77.6 | 1.5 |
|  | M40-M43:Deforming dorsopathies | 58.6 |  |  | F30-F39:Mood [affective] disorders | 41.2 |  |
|  | M80-M85:Disorders of bone density and structure | 50.5 |  |  | K70-K77:Diseases of liver | 36.1 |  |
|  | M15-M19:Arthrosis | 45.6 |  |  | F10-F19:Mental and behavioural disorders due to psychoactive substance use | 34.9 |  |
|  | G50-G59:Nerve, nerve root and plexus disorders | 34.7 |  |  | J40-J47:Chronic lower respiratory diseases | 31.6 |  |
| **3** | H53-H54:Visual disturbances and blindness | 49.4 | 1.7 | | K55-K63:Other diseases of intestines | 51.6 | 1.9 |
|  | L80-L99:Other disorders of the skin and subcutaneous tissue | 49.3 |  |  | K40-K46:Hernia | 41.9 |  |
|  | H49-H52:Disorders of ocular muscles, binocular movement, accommodation and refraction | 46.8 |  |  | K20-K31:Diseases of oesophagus, stomach and duodenum | 40.7 |  |
|  | L60-L75:Disorders of skin appendages | 45.2 |  |  | N20-N23:Urolithiasis | 35.3 |  |
|  | B35-B49:Mycoses | 44.5 |  |  | I80-I89:Diseases of veins, lymphatic vessels and lymph nodes, not elsewhere classified | 34.7 |  |
|  | H10-H13:Disorders of conjunctiva | 41.3 |  |  | N40-N51:Diseases of male genital organs | 29.9 |  |
|  | B00-B09:Viral infections characterized by skin and mucous membrane lesions | 41.2 |  |  | D10-D36:Benign neoplasms | 28.9 |  |
|  | L20-L30:Dermatitis and eczema | 39.2 |  |  | J30-J39:Other diseases of upper respiratory tract | 23.6 |  |
|  | N60-N64:Disorders of breast | 32.9 |  |  |  |  |  |
|  | D50-D53:Nutritional anaemias | 30.6 |  |  |  |  |  |
|  | H90-H95:Other disorders of ear | 26.2 |  |  |  |  |  |
|  | D10-D36:Benign neoplasms | 26.1 |  |  |  |  |  |
| **4** | K80-K87:Disorders of gallbladder, biliary tract and pancreas | 48.7 | 1.8 | | M45-M49:Spondylopathies | 70.3 | 1.9 |
|  | K40-K46:Hernia | 47.3 |  |  | M40-M43:Deforming dorsopathies | 68.2 |  |
|  | K70-K77:Diseases of liver | 43.0 |  |  | M15-M19:Arthrosis | 58.1 |  |
|  | K20-K31:Diseases of oesophagus, stomach and duodenum | 40.6 |  |  | G50-G59:Nerve, nerve root and plexus disorders | 53.7 |  |
|  | N20-N23:Urolithiasis | 35.4 |  |  | S80-S89:Injuries to the knee and lower leg | 51.0 |  |
|  | K55-K63:Other diseases of intestines | 35.2 |  |  | M20-M25:Other joint disorders | 35.7 |  |
|  | D50-D53:Nutritional anaemias | 27.7 |  |  | M05-M14:Inflammatory polyarthropathies | 34.3 |  |
|  | G40-G47:Episodic and paroxysmal disorders | 24.1 |  |  | M70-M79:Other soft tissue disorders | 27.0 |  |
| **5** | E10-E14:Diabetes mellitus | 77.0 | 2.1 | | H30-H36:Disorders of choroid and retina | 83.7 | 2.0 |
|  | I30-I52:Other forms of heart disease | 34.6 |  |  | I20-I25:Ischaemic heart diseases | 54.8 |  |
|  | E65-E68:Obesity and other hyperalimentation | 33.8 |  |  | I70-I79:Diseases of arteries, arterioles and capillaries | 54.1 |  |
|  | I10-I15:Hypertensive diseases | 32.3 |  |  | E10-E14:Diabetes mellitus | 50.9 |  |
|  |  |  |  |  | I30-I52:Other forms of heart disease | 38.8 |  |
|  |  |  |  |  | E65-E68:Obesity and other hyperalimentation | 30.4 |  |
|  |  |  |  |  | I10-I15:Hypertensive diseases | 27.8 |  |
| **6** | B25-B34:Other viral diseases | 83.9 | 2.2 |  | H10-H13:Disorders of conjunctiva | 57.0 | 1.9 |
|  | J09-J18:Influenza and pneumonia | 66.8 |  |  | B35-B49:Mycoses | 49.7 |  |
|  | A00-A09:Intestinal infectious diseases | 61.6 |  |  | J00-J06:Acute upper respiratory infections | 44.4 |  |
|  | T08-T14:Injuries to unspecified part of trunk, limb or body region | 28.4 |  |  | H53-H54:Visual disturbances and blindness | 41.7 |  |
|  | N30-N39:Other diseases of urinary system | 20.1 |  |  | L20-L30:Dermatitis and eczema | 37.0 |  |
|  |  |  |  |  | H60-H62:Diseases of external ear | 35.4 |  |
|  |  |  |  |  | L60-L75:Disorders of skin appendages | 34.8 |  |
|  |  |  |  |  | B00-B09:Viral infections characterized by skin and mucous membrane lesions | 34.4 |  |
|  |  |  |  |  | K00-K14:Diseases of oral cavity, salivary glands and jaws | 23.8 |  |
|  |  |  |  | | H90-H95:Other disorders of ear | 23.6 |  |
|  |  |  |  | | J30-J39:Other diseases of upper respiratory tract | 22.8 |  |
